# Key opportunities and challenges for the use of big data in migration research and policy

**DOI:** 10.14324/111.444/ucloe.000027

**Published:** 2021-10-27

**Authors:** Lydia H. V. Franklinos, Rebecca Parrish, Rachel Burns, Andrea Caflisch, Bishawjit Mallick, Taifur Rahman, Vasileios Routsis, Ana Sebastián López, Andrew J. Tatem, Robert Trigwell

**Affiliations:** 1 Institute for Global Health, University College London, London, UK; 2 Centre for Biodiversity and Environment Research, Department of Genetics, Evolution and Environment, University College London, London, UK; 3 Institute of Environment, Health and Societies, Brunel University, London, UK; 4 Centre of Public Health Data Science, Institute of Health Informatics, University College London, London, UK; 5 United Nations’ Displacement Tracking Matrix, International Organization for Migration, International Organization for Migration, Juba, South Sudan; 6 CU Population Center, Institute of Behavioral Science, University of Colorado Boulder Campus, Boulder, CO, USA; 7 Faculty of Environmental Sciences, Technische Universität Dresden, Dresden, Germany; 8 Health Management BD Foundation, Sector 6, Uttara, Dhaka, Bangladesh; 9 Adjunct Faculty, Department of Public Health, North South University, Dhaka, Bangladesh; 10 Department of Information Studies, University College London, London, UK; 11 GMV Innovating Solutions Ltd, HQ Building, Thomson Avenue, Harwell Campus, Didcot, UK; 12 WorldPop, School of Geography and Environmental Science, University of Southampton, Southampton, UK; 13 United Nations’ Displacement Tracking Matrix, International Organization for Migration, United Nations, London, UK

**Keywords:** big data, migration, cross-disciplinary research, policy, humanitarian, environment, displacement, climate change, health, data security

## Abstract

Migration is one of the defining issues of the 21st century. Better data is required to improve understanding about how and why people are moving, target interventions and support evidence-based migration policy. Big data, defined as large, complex data from diverse sources, is regularly proposed as a solution to help address current gaps in knowledge. The authors participated in a workshop held in London, UK, in July 2019, that brought together experts from the United Nations (UN), humanitarian non-governmental organisations (NGOs), policy and academia to develop a better understanding of how big data could be used for migration research and policy. We identified six key areas regarding the application of big data in migration research and policy: accessing and utilising data; integrating data sources and knowledge; understanding environmental drivers of migration; improving healthcare access for migrant populations; ethical and security concerns around the use of big data; and addressing political narratives. We advocate the need for careful consideration of the challenges faced by the use of big data, as well as increased cross-disciplinary collaborations to advance the use of big data in migration research whilst safeguarding vulnerable migrant communities.

## Introduction

**Figure 1:**
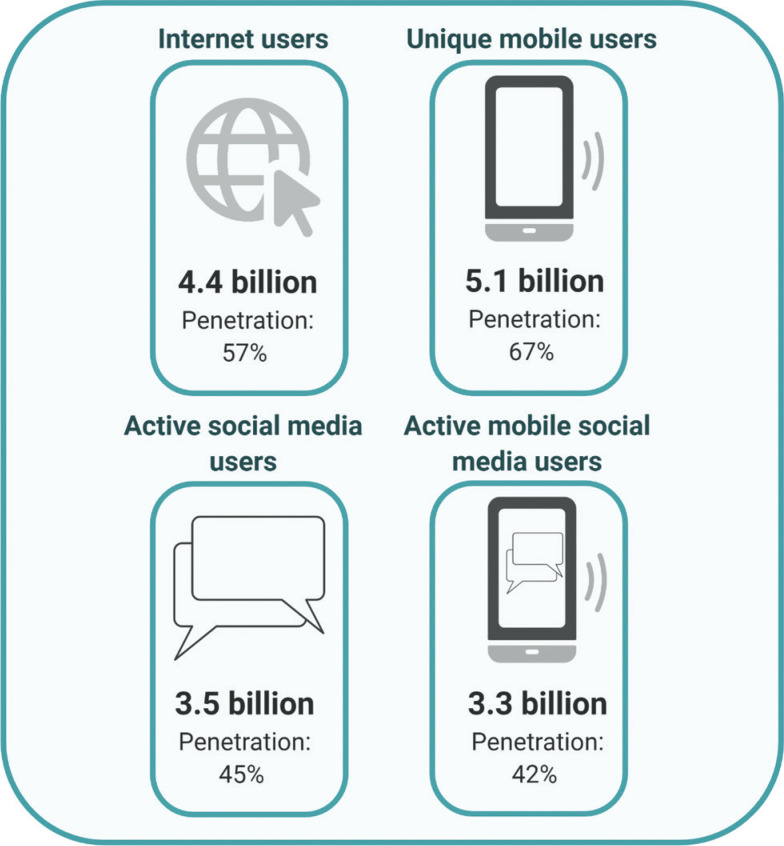
Key statistical indicators for global big data use. The number of users and portion of the population that has access (penetration) to the Internet, mobile phones, social media and mobile social media. Data were accessed via the Global Digital Report 2019 [5].

With the number of global refugees reaching the highest levels since the Second World War [1] and one billion migrants recorded in 2018 alone [2], human migration is high on the global political agenda. The University of London (UCL)-Lancet Commission on Migration and Health [2] and the United Nations Global Compact on Migration [3] have called for improved data to understand drivers of migration, target interventions and support evidence-based migration policy. The application of big data in migration research and policymaking has been proposed as a possible solution to help address these knowledge gaps [2,4]. Big data refers to large, complex data from varied sources, ranging from social media and mobile phone data (Fig. 1), to electronic health records and satellite data, and has the potential to provide new sources of information for migration research [6,7]. Previous studies have used big data to predict patterns of human movement during natural disasters [8] and track movement in near real-time [9], quantify migration at national scales [10–14], guide and evaluate humanitarian interventions [15] and examine the effects of human movement on disease transmission [16]. In addition, satellite-based Earth observation data has been used to map the relationship between environmental change and human movement [17,18], model subnational migration flows [19] and inform policy decisions [18,20,21]. Despite the immense opportunity big data can provide for migration research and policy, several challenges have hindered its widespread implementation [2,4].

In response to the call for increased collaboration [2] and improved research on ways to utilise big data sources in the field of migration [4], we participated in a cross-disciplinary workshop in London, UK on the 3rd July 2019, bringing together UN representatives, humanitarian non-governmental organisations (NGOs), policymakers and academics to facilitate knowledge exchange and identify the key opportunities and challenges for the implementation of big data in migration research. Here, we provide a summary of key discussion points identified in the workshop via presentations, panel discussions and break-out groups in which participants explored different topics and possible solutions. We provide major conclusions from the workshop supported by a review of the relevant literature to assist migration experts in deciding whether the use of big data is appropriate for their work and to stimulate discussion about the potential of this approach in aiding migration research and policy, and the needs of migrant populations globally. In particular, the outcomes of this workshop may provide a timely resource for the recently launched Lancet Migration, a global collaboration of migration experts that aims to address evidence gaps and drive policy change in the field [22]. Importantly, this workshop also tested a range of methods of collaborative working and identified challenges and opportunities in achieving a truly cross-disciplinary approach to migration research, an important but often neglected aspect in such a complex and politically charged topic as migration.

## Opportunities and challenges relating to big data

The aims of the workshop were to facilitate discussion on both the opportunities of big data but also the challenges, shortfalls and current ethical considerations surrounding big data and its uses. Whilst it is beyond the scope of a single workshop or article to find solutions to these major challenges, the workshop proved a valuable forum for raising awareness and nuanced discussion around these challenges which are often not fully recognised or addressed by researchers and practitioners alike when collecting or using big data as it relates to migration.

The format of the workshop consisted of keynote speeches, three panel discussions on the topics of 1) the role of big data in understanding the environmental drivers of migration, 2) the opportunities and challenges of big data in healthcare, and 3) the ethical considerations surrounding the use of big data in migration research and policy. The panel discussions were followed by group exercises which aimed to conceive structured, actionable solutions to a set of questions raised during the panel discussions. The resultant discussions and ideas were grouped into six topics which we summarise here and in Table 1.

**Table 1:** Key questions, challenges, opportunities and solutions for the use of big data in migration research and policy

Topics	Key research and policy questions	Potential challenges	Potential opportunities and solutions
Accessing and utilising big data	How can we improve access to big data sources? How can we enhance awareness of available data? How can we develop the expertise required to use big data across disciplines?	Issues of ownership and costs in accessing big data are significant barriers. If multiple mobile network operators are operating in a country, multiple data sharing agreements would be required [23]. Geographical biases in the type of data available. Poor awareness of fragmented data sources. Issues around the potential extraction of sensitive information contained in big data, therefore access arguably should be difficult for these data.	Opportunities for the development of centralised repositories of data such as The Humanitarian Data Exchange [24] to promote collaboration and knowledge-sharing. Opportunities for partnerships across different sectors to improve access to available data and technologies. Capacity building projects across disciplines such as the UNECE Big Data Sandbox [25] are needed to support big data use for migration research.
Integrating data sources and knowledge	How can we produce more detailed and recent migration statistics using big data? How can we best integrate data from different sources? How do we manage fragmented data sources across varied spatial and temporal scales? How can we develop a collaborative cross-disciplinary approach to address the challenges in the field of migration?	Complex analyses are required to account for multiple biases in different datasets. Lack of awareness and big data expertise in humanitarian sector has led to its slow adoption.	The use of big data sources combined with traditional survey-based research can reveal important aspects of migration that are often not captured (e.g., CDRs reveal short-term migration patterns) [26]. Potential for methodological innovation for the integration of different data and the development of a ‘gold standard’ to estimate migration using near-real-time big data. Opportunities for cross-disciplinary ‘Data Collaboratives’ [27] to enable data exchange and help address complex problems.
Understanding environmental drivers of migration	How can big data be used to assess the ongoing impact of climate change on migration? Can big data help to identify populations that are vulnerable to environmental change? How can big data be used to predict mass migration events due to environmental change?	Satellite-based environmental data is often at coarse geographic scales which are not suitable to inform sub-national policies and actions. There may be discrepancies between the assumed environmental drivers of migration perceived from satellite data analyses and the self-reported drivers of migration.	Opportunity to address the lack of evidence on the relationship between migration and environmental change and to help to define the term ‘environmental migrant’ that is needed for policy action. Potential to reveal important aspects of migration associated with extreme weather events that are often not captured with traditional data. Potential to unpick socioeconomic factors that may be limiting people’s ability to migrate as an adaptation strategy to environmental change. Opportunities to inform policies that will improve resilience and support migration associated with environmental change.
Improving healthcare access for migrant populations	How can big data be used to address the immediate health needs of displaced persons in camps? How can big data help us learn more about undocumented migrants, their health and healthcare needs? How can big data be used to implement evidence-based health interventions?	Big data approaches in healthcare risk jeopardise the protection from state surveillance that is currently provided to vulnerable communities such as undocumented migrants.	Big data can help to understand the impact of migration on the transmission of infectious diseases. Potential to support on-the-ground activities, helping to address the immediate health needs of displaced persons and to predict disease outbreaks. Potential for big data to improve equitable healthcare access for underrepresented communities, such as undocumented migrants and vulnerable groups. Opportunities to improve the specific healthcare needs of migrants with settled status. Applications for big data in identifying differences in patient responses to treatments and tailoring healthcare to the specific needs of individuals.
Ethical, privacy and security concerns	What is meant by ethics in the context of big data in migration research? Who benefits from the use of big data (migrants at individual or community level, academic community, policymakers)? How do power imbalances influence the use of big data? How can we achieve ethical data usage?	Big data analytics may lead to the introduction of stakeholders which have varied motivations. Biases in big data may follow through into policies, propagating stereotypes and discriminatory practices, or else continuing to underserve invisible groups. There is often focus on the legal aspects of data protection for personal data rather than the potential negative impacts on affected vulnerable groups. Big data use in migration can promote the power imbalance between those seeking data and those the data is being extracted from. There is no legal enforcement of guidelines for safe and ethical data management in humanitarian situations.	Ethical considerations and safeguarding practices are required when involving varied stakeholders in big data analytics The use of big data in migrant research requires the clear stating of assumptions and methods to correct for biases to prevent the propagation of biases in policy. Ethical considerations and safeguarding practices are required when involving varied stakeholders in big data analytics. Researchers and decision-makers must critically examine and justify why they require the use of big data and whether this is what all parties, particularly migrants, would want.
Addressing political narratives	How can we prevent the use of big data for the discrimination of certain populations? What role could big data have in addressing the negative political narratives around migration?	Big data use may lead to lack of nuance behind the motivations for migration, potentially distorting the narrative behind migration patterns. Ongoing anti-immigration rhetoric means it is imperative that big data is not used to further discriminate against migrant communities or to target certain populations.	Opportunity to help to address negative political narratives and to support inclusive and fair migration governance as seen with the Sentinel project, which works to counter the spread of misinformation and antimigrant rhetoric [28]. Designing strong, cross-disciplinary communication tactics to support maximum impact of evidence. Opportunity to help to challenge public and media perceptions of global migration which has been shaped by misinformation due to paucity in migration data.

### Accessing and utilising big data

The first topic focused on the access, awareness and expertise required for big data use. The application of big data is often hindered by the fact that many big data sources such as mobile phones, Internet-based platforms and other digital devices are managed by private companies who collect the data for business purposes. Therefore, costs associated with accessing big data and issues of ownership are significant barriers to its use [29]. Big data generation will vary geographically and may be reduced in many high mobility contexts where infrastructure (i.e., cell towers, Wi-Fi connection and electronic bank transfer services) is less established. Specific to the use of mobile phone call detail record (CDR) data, the presence of multiple mobile network operators in a country means that multiple data sharing agreements would be required [23]. In addition, there are significant issues around the potential extraction of sensitive information contained in big data [30,31] and data sources are often fragmentated across disciplines which reduces the awareness of available datasets [32]. Accounting for multiple biases and the complex analyses required to interpret the data are further examples of methodological difficulties associated with the use of big data [7,33].

Workshop discussions highlighted the importance of understanding how, why and when the data were collected to identify potential gaps and biases, therefore ensuring it can be used effectively. There is great need for more centralised repositories of data, projects and publications such as The Humanitarian Data Exchange [24], to promote knowledge-sharing, collaborations and inform evidence-based programming. Increased partnerships between governments, international agencies, civil society and the private sector are also required to improve data access and ensure the optimum exploitation of available data and technologies. Furthermore, capacity building in countries or organisations with an interest in big data analysis is needed to support cross-disciplinary research and improve specialist knowledge in certain regions. This could be achieved via collaborations with relevant partners and agencies such as has been demonstrated with the United Nations Economic Commission for Europe’s (UNECE) Big Data Sandbox, which provided a platform for statistical organisations to collaborate and learn to use big data analytics [25]. However, there may be ethical considerations for private–public partnerships. For example, published commentaries have voiced fears over the partnership between the UN’s World Food Programme and the data analytics company Palantir, which may have serious consequences for the privacy and security of aid recipients due to the company’s links to United States (US) intelligence agencies [34].

### Integrating data sources and knowledge

**Figure 2:**
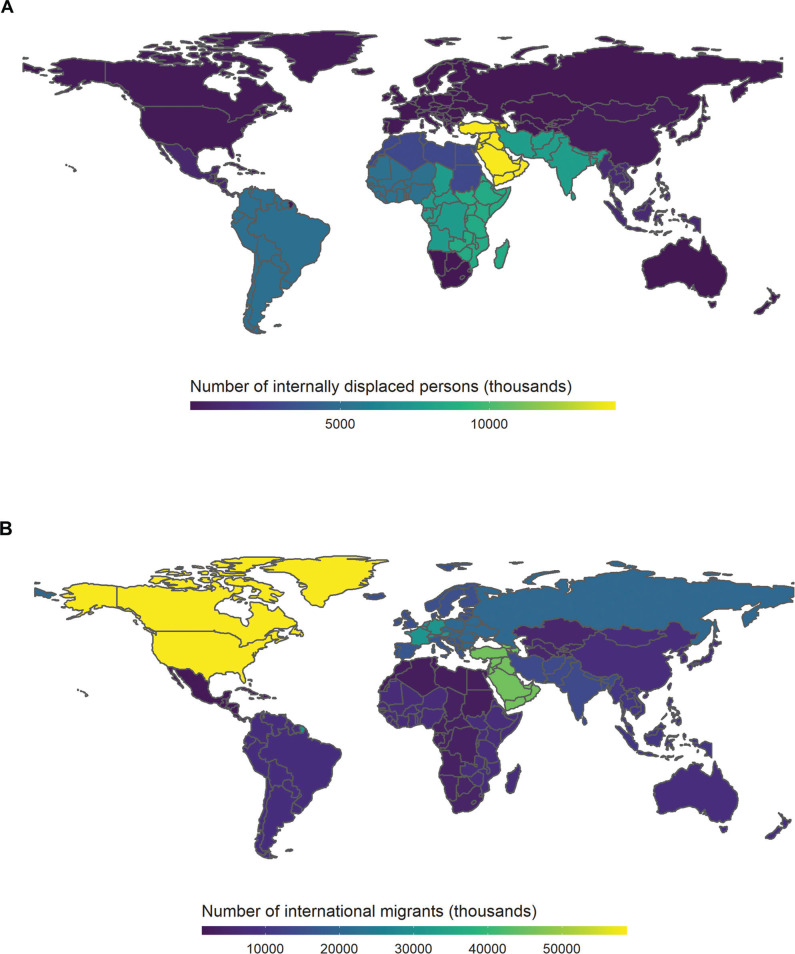
Global human migration by subregion. A) Number of internally displaced persons (from conflicts and disasters) in 2020 ranging from 22,000 in Eastern Europe (dark blue) to 14,200,000 in Western Asia (yellow); data were accessed via the Internal Displacement Monitoring Centre’s Global Internal Displacement database [35]. B) Number of international migrants in 2019 ranging from 1,542,000 in the Caribbean (dark blue) to 58,647,000 in North America (yellow); data were accessed via the UN Department of Economic and Social Affairs, Population Division database [36]. The legends for both graphs show the number of migrants in thousands.

The second topic concerned the integration of data and knowledge across disciplines. The main source of data for migration statistics originates from traditional methods such as household surveys recorded using local scales and national population estimates, as well as data on forced displacement collected through key informant networks [4] (Fig. 2). Big data sources have the potential to complement traditional data and address significant spatial and temporal gaps via updating migration statistics in an accurate and low-cost way [4,11]. For example, analysis of CDR data can be used to replicate national internal migration statistics and complement outputs from censuses [11]. However, integrating migration data from traditional methods with varied sources of big data requires new methodology that considers complex interactions over differing geographical and temporal scales. The quality of various data sets (e.g., demographic biases present in social media datasets) remains an unresolved challenge in teasing out comprehensive, policy-relevant results. Validating estimated migration using near-real-time big data is also problematic, with no trusted ‘gold standard’ currently available [37]. The slow adoption of big data analyses in the humanitarian sector is partly due to a lack of expertise in how to apply these approaches in operational settings [38]. Workshop participants discussed the need to bridge the gap between experts on the ground collecting the data via traditional methods and big data analysts via increased transdisciplinary training and collaborations. A recent workshop hosted by the International Organization for Migration (IOM) and the German Federal Foreign Office concluded that ‘greater cooperation and engagement among stakeholders’ both within and external to the migration sector are required to inform decision making [39]. If we are to integrate different data sources effectively, a collaborative cross-disciplinary approach is required to ensure we understand the data and how they can be used to deepen our understanding of the drivers and impacts of migration. This approach is practiced in ‘Data Collaboratives’; collaborative projects in which different sectors including private companies, research institutions and government agencies collaborate to enable data exchange and help solve public problems [27]. NetHope is an example of a data collaborative project which has helped to integrate data sources and produce maps of connectivity sites across Puerto Rico to assist in delivering aid in the aftermath of Hurricane Maria [40].

### Understanding environmental drivers of migration

The third topic considered the use of big data in understanding and identifying environmental drivers of migration such as natural hazards and climate change [41], for example, via remote sensing. Currently, there is no internationally agreed definition for ‘environmental migrant’ despite it being required to collect long-term data and guide the policies of governments and international agencies. The IOM have proposed a broad working definition [42] which importantly considers that environmental migration might be triggered both by sudden-onset events [43], such as earthquakes and cyclones, and slower environmental change processes, such as desertification and sea-level rise. In the context of slow-onset events and gradual environmental change, the effects on migration are often difficult to quantify as it can be hidden behind more immediate socioeconomic drivers such as poverty or political processes [44]. There are opportunities for the application of big data to unpick these different drivers and to help to define the term ‘environmental migrant’. A further application could be in helping to quantify the effect of environmental change on the trapping of individuals or communities, usually due to rising poverty barriers which impede mobility. Human mobility can improve resilience and attenuate the negative outcomes of environmental degradation, but poverty, disability and social exclusion may limit people’s ability to resort to migration as an adaptation strategy [45].

Despite the significant attention that environment-induced human migration has received [46], evidence on the relationship between migration and environmental change is limited. When used in combination with traditional datasets, big data has the potential to identify hot spots of environmental change and exposed populations that may be affected by the change and therefore liable to migrate, or to become trapped. Satellite data is a particularly valuable resource in analysis of environmental drivers as it enables the systematic, consistent and accurate monitoring of areas (even if remote or inaccessible) that are affected by anthropic or natural hazards. Indeed, satellite-based technologies are key to analysing observable effects of climate change effects and even predicting environmental-led migration [47–52]. Furthermore, a study combined satellite data and CDR data to quantify the incidence, direction and duration of flood-driven migration, revealing important short-term (hours–weeks) aspects of migration associated with extreme weather events that are often not captured with traditional survey-based research [18]. There is great potential for big data to help in understanding and addressing environment-related displacement and to inform policies that will improve resilience to environmental change and support migration that is required to improve the health and livelihoods of vulnerable people. In such examples, improved data on both environmental change (as a driver of migration) and of migration itself (such as displacement following a natural hazard) cannot ‘solve the problem’ of forced environmental migration but can inform interventions such as aid as well as discussions between affected communities and stakeholders in devising context-appropriate solutions for the future.

One of the most valuable aspects of satellite-based analyses is the capability for retrospective analysis which is required to detect changing patterns across space and time and to inform predictions. However, a recent review stated that current initiatives do not exploit the full possibilities of satellite-based earth observation in migration with a lack of services offering the systematic flow of detailed information to researchers, managers and migration analysts [53]. One of the main gaps identified is that consolidated satellite-based monitoring systems currently work at regional scales, providing a geographic resolution that is often too coarse to understand the specificities of how particular communities are affected. These datasets are thus unable to reliably inform the design, implementation and monitoring of sub-national policies. Indeed, reported discrepancies between the assumed environmental drivers of migration perceived from satellite data analyses and the self-reported drivers of migration (e.g., see [54]) underline the importance of including information on the lived experiences of migrants to inform actions.

### Improving healthcare access for migrant populations

The fourth area of discussion focused on the potential for big data to enhance migrant health via improved disease outbreak preparedness, identification of vulnerable groups, increased access to healthcare and by informing evidence-based health interventions. Many studies have demonstrated the potential for big data to understand the impact of migration on the transmission of infectious diseases such as dengue [16], malaria [19,55] and cholera [56] at a national scale. These analyses highlight the potential benefit of big data use to improve preparedness and mitigation efforts for disease outbreaks. This may be particularly useful when supporting on-the-ground activities by helping to predict potential disease outbreaks for displaced persons. A recent collaboration between the IOM and the mobile operator data analytics organisation Flowminder, is combining IOM Flow Monitoring Registry surveys with CDR data to gather anonymous information about people on the move at key transit points to inform public health interventions for the coronavirus (COVID-19) pandemic [26]. A further application of big data in humanitarian settings is the use of satellite data to map refugee settlements [21], which can be used to ensure healthcare access for displaced persons.

A perennial issue in equitable healthcare access is identifying and addressing the needs of invisible communities, such as migrants, particularly those with an undocumented status [2]. Shortfalls with traditional datasets and data collection processes such as semi-structured interviews and surveys means there is limited information on undocumented migrants and vulnerable groups (i.e., unaccompanied children, people with disabilities and members of the lesbian, gay, bisexual, transgender and intersex (LGBTI) community). There is great potential for big data to address the paucity of information on these groups, their health, access to healthcare and differing healthcare needs. In addition, it was suggested that the healthcare needs of migrants settled in countries such as the UK could also be improved by big data analysis, for example, via a general migrant longitudinal study such as the cohort studies performed by the UK Economic and Social Research Council [57]. There are also vast applications for big data in implementing evidence-based health interventions that need to be explored, specifically in identifying differences in patient responses to treatments and tailoring healthcare to the specific needs of individuals [58].

The COVID-19 pandemic has received widespread support for the use of big data in disease surveillance systems globally [59]. Such approaches have had various levels of success at curbing infection rates, particularly crucial for vulnerable persons (which could include some migrant communities). However, if the use of such big data approaches in healthcare became common place, it may cost many the protection that invisibility currently offers, with many communities such as undocumented migrants fearing disproportionate effects of state surveillance [60]. Such ethical conundrums remain unresolved and often underexplored. At best, it is likely that big data should not be seen as a perfect solution but as one optional tool within a wider social toolkit which retains traditional and non-digital interventions.

### Ethical, privacy and security concerns

The fifth topic focused on ethical, privacy and security concerns regarding the use of big data in migration research. This topic proved to be cross-cutting, with themes re-emerging across other topics. Principally, a recurrent question arose about whether it is appropriate to collect and/or use sensitive data in the pursuit of greater understanding for researchers and policymakers. The collection of personal data including migrant status is a contentious issue. There are concerns that information on personal migration status may create or increase existing discriminatory practices in society such as the provision of healthcare and access to state funds, or that mobile tracking devices may be used against a migrant to forcibly return them to a previous location [2]. Additionally, various sources of big data (such as social media) are consumed differently based on geography, demography and access. As such, any analysis of these datasets will carry these biases, which may follow through into policies. This may result in policies which propagate stereotypes and discriminatory practices, or else continue to underserve invisible groups (e.g., those not engaged with social media or with smaller social networks) [37].

Ethics in the context of big data in migration may be considered in several ways. Firstly, it may relate to the way in which the research is conducted and whether there has been consideration for data privacy and security. Secondly, it could refer to decision-making regarding migrants with consideration for their lived experience, especially in humanitarian situations. A recent report on migration noted that discussions on ethics often focus on the legal aspects of data protection rather than understanding how the results of analyses may detrimentally impact affected populations and counter the humanitarian principles to ‘do no harm’ [39]. Furthermore, data protection measures are often focused on personal data (e.g., General Data Protection Regulation in the European Union) and do not consider group data protection needed to work with vulnerable groups [61].

It is important to consider who benefits from the use of big data sources in migration research. At the individual level, migrants may not wish for additional data to be gathered about them and may perceive no benefits of the process [62]. However, at the community level, such data and analysis may help to address the health needs of migrants more generally. Certainly, there will be benefits to the academic community seeking to study migrant health needs and to decision-makers seeking evidence-based solutions. Pursuit of these research and policy goals can result in overlooking the individual rights and raise ethical issues for many vulnerable people [62]. Furthermore, forcibly displaced people fleeing persecution may have little trust in authorities and therefore be less willing to seek healthcare or consent to having their data collected. This creates a barrier for healthcare professionals, humanitarian workers and researchers who wish to respect the rights of the individual, whilst deriving a better understanding of migration pathways and healthcare needs. Workshop discussions highlighted the power imbalance between various parties involved; those seeking data including governments and academics often from the global North with inherent biases and power, and those the data is being sought from who are often vulnerable persons in precarious or dangerous situations, many originating from the global South [63]. Evidence of this power imbalance is seen with ‘high-risk experiments’ with new technologies such as the use of Canada’s automated decision-making technology in immigration and refugee applications [64] which often lack regulation. Even after applying advanced safeguarding practices and aggregated outputs, researchers may still be reluctant to apply big data analysis for migration research as policy makers often have their own agendas and may use the methods and deliverables in ways not intended or anticipated by the research authors.

Another established concern regarding the ethical deployment of big data in migration research is the focus on pattern-based analyses, rather than on achieving a conceptual interpretation and critical analysis for why behavioural trends might emerge and under what set of circumstances and assumptions [65,66]. Indeed, the limitations and bias of big data remains an under-explored aspect in the discourse around ethical implications of big data. The COVID-19 pandemic provides a pertinent example of how the lack of data from vulnerable communities such as refugees or people on the move has led to the underrepresentation of these communities in the narrative and political responses of the pandemic [67].

### Addressing political narratives

The final topic concerned the role of big data in high level political narratives around migration. With ongoing antimigrant rhetoric existing at all levels of government and society, migrant research has the opportunity and mandate to address such political narratives. There are many examples of authorities treating migrants as political pawns or as statistical figures [68]. Therefore, it is imperative that big data is not used to further discriminate against migrant communities or to target certain populations, but rather to support inclusive and fair migration governance. This can be particularly problematic with big datasets, where assumptions have been made throughout the data collection and analysis process. For example, in 2017 the UK data analytics company CGI together with the Dutch statistical agency CBS, conducted a study into migration forecasting of people in Syria using Twitter data [66]. The study required a set series of assumptions to be made about Twitter content (more specifically its English translation) and was unable to consider the context within which tweets were posted. This case study demonstrates how the promotion of big data can reify such assumptions and distort the original meaning or intent behind an individual’s migration decisioning, as well as pervert the aggregate narrative behind migration patterns [66]. Conversely, a positive example discussed within the workshop was the Sentinel project; an NGO that works to gather and disseminate ‘trusted’ information to local people and governments in order to counter the spread of misinformation, antimigrant rhetoric and to prevent resultant hate crime and genocides [28]. Participants also deliberated whether the increased evidence provided by big data would be instructive and powerful enough to overcome political and social biases associated with the topic. Given the highly political nature and high stakes of migration policy for migrants as well as for governments and the international community, more evidence may alone be insufficient to achieve multilateral, progressive action. Therefore, it is worth considering other factors contributing to the political discourse and designing strong, cross-disciplinary communication tactics to support maximum impact of evidence. Furthermore, it is worth considering how paucity in migration data has helped to shape public and media perceptions of global migration patterns to date, and whether big data could be used to address these perceptions.

## Discussion

The application of big data in migrant research shows much promise in addressing the current gaps in knowledge. Big data sources can help to update internal migration statistics by addressing the significant gaps in quantity and quality of data collected from traditional methods [4,11]. When combined with field-level data derived from household surveys and key-informant networks, big data can be used to detect how sudden onset natural hazards and gradual environmental change (e.g., desertification and sea-level rise associated with climate change) impact migration patterns. This can help to inform planning and scenario building, as well as contributing to a more comprehensive definition of ‘environmental migrant’ which is critical for migration policy within the context of ongoing environmental change. In addition, it has a potential application in considering the differing healthcare needs of migrants as well as identifying vulnerable populations unable to migrate due to environmental change. There is also scope for big data to inform evidence-based health interventions for migrant populations in everyday and emergency (including displacement) settings. Yet despite the vast opportunities that big data present, there are some important areas to consider before using these varied and complex data sources. Increased cross-disciplinary partnerships are required to improve data access, knowledge-sharing and capacity building across sectors and regions. In addition, a collaborative cross-disciplinary approach is required to ensure the different datasets are understood and to develop new methodologies to integrate data sources and identify complex interactions that influence how and why people are moving. Furthermore, the reported lack of agreement within the humanitarian sector on how migration modelling should be applied needs to be addressed so analyses can be effective [39]. It is important to challenge the assumption that big data is always a suitable and insightful tool to use in research for migration policy. Whilst tempting to consider big data the magic bullet, it may not always be appropriate or may need to be used in conjunction with traditional methods.

International legislation is required to sufficiently address how migrant data should be collected and used to ensure ethical conduct by data gatherers and owners and the safeguarding of human rights, particularly in sensitive migration contexts [69]. The United Nations Development Group [69] and Office for the Coordination of Humanitarian Affairs [70] provide guidelines for safe and ethical data management in humanitarian situations; however, there is no legal enforcement of these practices. Although researchers would like quicker and easier access to data, workshop discussions challenged whether the process should be hastened, suggesting that administrative obstacles force researchers to duly consider whether additional data is necessary and beneficial to the current state of knowledge, given the risks and trade-offs that must be made. A key output of the workshop was a consensus that researchers and decision-makers must first ask why they require additional data and whether this is what all parties, particularly migrants, would want. As well as data protection issues, it is also imperative to understand the potential harmful impact of analyses on vulnerable migrant populations [39]. It is especially important that big data is not used to further discriminate or target migrant populations considering current antimigrant political narratives.

In pursuit of cross-disciplinary collaboration, the workshop brought together a range of representatives from the UN, government, humanitarian agents and academics from a range of backgrounds. Cross-sector engagement in the workshop was difficult to achieve, which may be due to differences in the objectives of different sectors, as well as the language of engagement used. For instance, humanitarian organisations were particularly difficult to engage, and it is thought that this was due to both a shortage of networks linking academia and humanitarian organisations as well as differences in short-, medium- and long-term needs and objectives of the two sectors. We trialed different methods to stimulate interdisciplinary work including the use of business canvases [71] to explore and present solutions to questions. This approach was useful for stimulating debate within the groups and producing well-considered outputs. However, future interdisciplinary events would benefit from the development of methods that consider the language styles and information sharing techniques of different disciplines and thus facilitate effective communication and knowledge-sharing [4,39,72,73]. The workshop also elucidated the extent to which the direction of conversation is steered by which actors are engaging in the conversation. For example, actors engaging with migrants directly (i.e., service providers) focus on practical implications. This serves as a pertinent reminder of the importance for academics to create and utilise diverse networks as well as the need for actors with gatekeeping power to exercise due diligence and engage a wide range of stakeholders and interest groups in discussions. Overall, the workshop highlighted the benefits of cross-disciplinary work, enabling the identification of key topics from a variety of angles and providing meaningful and effective outputs. Furthermore, we hope this workshop assists in cultivating a future transdisciplinary approach to migration research, whereby there is a move beyond the collaboration of individual disciplinary perspectives to develop curriculum integration that organises knowledge production in the context of real-world problems [74].

## Data Availability

Data sharing not applicable to this article as no datasets were generated or analysed during the current study.
